# Hippocampus- and neocortex-specific deletion of *Aeg-1* causes learning memory impairment and depression in mice

**DOI:** 10.1038/s41419-025-07508-0

**Published:** 2025-03-23

**Authors:** Ya-he Wang, Ning Zhou, Pan-pan Wan, Xin-tong Li, Chun-yang Yu, Jinjiang Chou, Zong-yi Feng, Lian-xiang Zhang, Juan-juan Li, Bao-cong Yu, Zhen-ning Tang, Kun-mei Liu, Le Guo

**Affiliations:** 1 Ningxia Key Laboratory of Cerebrocranial Disease, Ningxia Medical University, Yinchuan, Ningxia 750004 China; 2 Department of Cell and Molecular Biology, Karolinska Institutet, Solnavägen 9, 17165 Stockholm, Sweden; 3 Department of Surgical Oncology, General Hospital of Ningxia Medical University, 750004 Yinchuan, Ningxia PR China; 4 Ningxia Key Laboratory of Clinical and Pathogenic Microbiology, General Hospital of Ningxia Medical University, Yinchuan, Ningxia 750004 China; 5 College of Laboratory Medicine, Ningxia Medical University, Yinchuan, Ningxia 750004 China

**Keywords:** Hippocampus, Spine structure, Cortex

## Abstract

Astrocyte elevated gene-1 (AEG-1) has been characterized as an oncogene promoting the progression of various tumors. The role of AEG-1 in neurological diseases was highlighted by recent researches. However, the physiological function of AEG-1 remains elusive. Our study aimed to investigate the physiological role of AEG-1 in the central nervous system by generating a mouse model with specific deletion of *Aeg-1* in the hippocampus and neocortex (*Aeg-1*^fl/fl^Cre^+^ mice). Behavioral assessments revealed that *Aeg-1* deficiency caused impaired learning and memory capabilities in juvenile and adult mice. Depressive-like behaviors were also observed in *Aeg-1*^fl/fl^Cre^+^ mice. Gene Ontology (GO) enrichment analyses indicated that AEG-1 was involved in the neuronal morphogenesis. Interestingly, *Aeg-1* knockout was irrelevant to the neuron loss but reduced the dendritic length and the dendritic spines density in hippocampus. Electrophysiological analyses showed a decreased response of paired-pulse facilitation (PPF) and a compromised efficiency of excitatory synaptic transmission following *Aeg-1* deletion in hippocampus. In conclusion, our findings suggest that *Aeg-1* deficiency in the hippocampus and neocortex leads to learning and memory impairments and depression in mice, which is mediated by the abnormalities of neuronal morphology and the impaired synaptic functions.

## Introduction

Astrocyte elevated gene-1 (AEG-1), also known as Metadherin (MTDH) or Lysine-Rich CEACAM1 co-isolated (LYRIC), is firstly identified in the astrocytes of HIV-infected patients with Alzheimer’s disease [1]. Accumulated studies have revealed that AEG-1 is dysregulated in many malignant tumors and the dysregulated AEG-1 is associated with the proliferation, migration, invasion, angiogenesis and chemotherapy resistance in tumors [2]. In addition, AEG-1 displays critical functions in non-cancer diseases, especially in nervous system diseases including but not limited to neurodegenerative diseases, migraine, amyotrophic lateral sclerosis (ALS) and epilepsy [3, 4].

Current studies mainly focus on the regulatory mechanism of AEG-1 in astrocytes during the progression of neurological diseases due to AEG-1 brain astrocyte origin [5]. Up-regulation of AEG-1 induces YY1 activity, which subsequently represses CBP, one of the coactivators of EAAT2 promoter. Therefore, the glutamate uptake mediated by EAAT2 in astrocytes is restrained, leading to increased glutamate excitotoxicity and induction of neuronal cell death (Fig. S1) [4]. Furthermore, AEG-1 is associated with astrocyte activation, which promotes reactive gliosis in vivo and mediates astrocyte migration after injury [6]. Interestingly, recent studies gained the conclusions that the regulatory role of AEG-1 is not limited in astrocytes, but also in neuron [5]. AEG-1 is expressed in motor neurons and enhances the viability of motor neurons in amyotrophic lateral sclerosis (ALS) [7]. In addition, AEG-1 is capable of protecting nigral dopaminergic (DA) neurons against apoptotic damage. Clinical data showed that AEG-1 is suppressed in postmortem nigra of Parkinson’s disease (PD) patients compared to the age-matched controls [8]. Notably, AEG-1 is elevated in astrocytes whereas it is repressed in neurons during the progression of neurological diseases [5].

However, the role of AEG-1 in normal physiological conditions is still largely unknown due to the limited evidence. High expression of AEG-1 was observed in the neurons but not in the normal glial cells from adjacent noncancerous tissues (ANT) [9]. Additionally, AEG-1 is involved in neural development during the embryonic period as AEG-1 level decreases at the embryonic day 18.5 compared to the day 10.5 [10]. A recent study indicated that AEG-1 is expressed in both hippocampus and cortex [11]. Since hippocampus is critical for learning and memory [12, 13], our study will utilize a transgenic mouse model rendered specific *Aeg-1* loss in hippocampus and cortex to clarify the role of AEG-1 in learning and memory maintenance in normal physiological conditions.

## Materials and Methods

### Animals

Emx1-IRES-Cre mice (Stock No. 0056289) were purchased from the Jackson Laboratory. Wild-type C57BL/6 J mice were obtained from the Experimental Animal Center of Ningxia Medical University. Genetically modified mice (heterozygous *Aeg-1*^fl/wt^, homozygous *Aeg-1*^fl/fl^, and hippocampus-and neocortex-specific *Aeg-1* knockout *Aeg-1*^fl/fl^Cre^+^) were bred on a C57BL/6 J background. With the exception of breeding experiments, all animals involved in the subsequent experimental procedures were male. The animals in this study were assigned to different experimental groups through randomization. Mice were housed in ventilated cages with free access to food and water under controlled conditions (24 ± 0.5 °C, 40–70% RH, 12 h light/dark cycle). Experiments were approved by Ningxia Medical University’s Ethics Committee, adhering to animal ethics laws and minimizing suffering.

### Generation of Transgenic Mice

#### Generation of *Aeg-1*^fl/wt^ Mice with Inserted loxP Sites

*Aeg-1* was cleaved at introns 2–3 and 6–7 by Cas9/sgRNA. LoxP sites were inserted into introns 2–3 and 6–7 subsequently by introducing a loxP donor. The constructed Cas9/sgRNA plasmid and loxP-donor were microinjected into mouse zygotes followed by implantation into pseudopregnant mice. The obtained embryos were developed into full-term pups. Positive *Aeg-1*^fl/wt^ F0 founder mice were identified by PCR, sequencing, and southern blot analysis. *Aeg-1*^fl/wt^ F1 offspring was generated by crossing *Aeg-1*^fl/wt^ F0 mice with C57BL/6 J mice. SgRNAs were listed in Table S1.

#### Generation of Hippocampus-and Neocortex-Specific *Aeg-1* Knockout *(Aeg-1*^fl/fl^Cre^+^) Mice

We intercrossed *Aeg-1*^fl/wt^ F1 mice to obtain homozygous *Aeg-1*^fl/fl^ mice, which were subsequently crossed with Emx1-IRES-Cre mice to generate *Aeg-1*^fl/wt^Cre^+^ mice. The *Aeg-1*^fl/wt^Cre^+^ mice were intercrossed to generate homozygous *Aeg-1*^fl/fl^Cre^+^ mice. The homozygous genotype of *Aeg-1* deletion in hippocampus and neocortex was verified by PCR.

### DNA extraction, PCR, and sequencing

The insertion of loxP and other sites was confirmed in newborn mice by PCR genotyping and sequencing of tail DNA. Table S2 lists the primers used. PCR conditions included initial denaturation at 98 °C for 30 s, followed by 35 cycles of 98 °C for 10 s, annealing at 50–60 °C for 28 s, and extension at 72 °C for 28 s, with a final extension at 72 °C for 90 s. PCR products were stored at 4 °C and separated on a 2% agarose gel at 120 V for 30 min. Gel bands were visualized using an electrophoresis gel imaging system (JS-860B M7150, Peiqing Technology).

### Southern blot

DNA from *Aeg-1*^fl/wt^ F0 mice was digested with *Sac* I to produce 11.169 kb fragments, which were resolved on a 1% agarose gel and transferred to a nitrocellulose membrane. After UV cross-linking for 30 min, the membrane was hybridized with specifically labeled DNA probes (listed in Table S3). Hybridization signals were visualized using a fluorescence imager (Amersham Imager 600, GE Healthcare) to confirm the binding between the labeled probes and target DNA fragments.

### Stereotaxic injections

60-day-old *Aeg-1*^fl/fl^Cre^+^ mice were anesthetized with continuous isoflurane (3% induction, 1% maintenance) and fixed on a stereotaxic apparatus (RWD Life Science Co., Ltd, Shenzhen, China). The skull was exposed, and the meninges were removed with H_2_O_2_. The bregma served as the reference for determining injection coordinates in the MoDG (AP, −1.79 mm; ML, ±1.25 mm; DV, −1.75 mm) and S1BF (AP, −1.79 mm; ML, ±2.75 mm; DV, −1.40 mm). Virus (1 μL AAV9-Mtdh (109903-2), from Shanghai Genechem Co., Ltd, Shanghai, China) was injected at 0.2 μL/min, with the electrode left in place for 9 min post-injection. The wound was sutured, and behavioral experiments were conducted three weeks later.

### Behavioral experiments

The mice were acclimated in the behavioral testing room one day before the experiments. The Intellicage system (TSE Systems, Thuringia, Germany) was employed to evaluate the learning and memory abilities in *Aeg-1*^fl/fl^Cre^+^ mice. Tail suspension and forced swimming tests were conducted to assess emotional changes in *Aeg-1*^fl/fl^Cre^+^ mice.

#### Intellicage system test

Prior to placement in the Intellicage system, each mouse was implanted with an RFID transponder chip in the neck region. The test procedure was as follows: (A) Free exploration (3 days): With all four corners’ doors open, allowing mice free access to all areas and water. (B) Nose-poke adaptation (3 days): Doors to all corners were closed and only opened when a mouse’s nose touched the internal sensor, granting the mouse access to water. (C) Place learning (6 days): With doors to all corners closed, the correct corner, designated as the sole nose-poke-activated door access to water, was the one opposite to the most frequently visited corner during the adaptation phase. The remaining three corners were incorrect. (D) Reversal place learning (6 days): With new correct corner opposite to the correct corner in phase C, and the remaining three were incorrect. Cognitive function was assessed by analyzing data on visits, nose-pokes, and drinking during the place learning and reversal place learning phases.

#### Tail suspension test

The end of the mouse’s tail was attached to the hook of the tail suspension device (RWD Life Science Co., Ltd). An infrared camera connected with a computer was positioned to record the experiment. The behaviors of mice were recorded in the 5 min test duration and the percentage of immobility and struggling was analyzed using the SMART 3.0 (Panlab SL Inc., Barcelona, Spain, supported by RWD Life Science Co., Ltd).

#### Forced swimming test

The swim tank (RWD Life Science Co., Ltd) was filled with water (approximately 22 °C) to a depth of 20 cm. The method of recording and analyzing the experimental data followed the same procedure as Tail Suspension Test.

### RNA-seq analysis

Experimental mice were euthanized following anesthesia by inhalation of 2% isoflurane. The hippocampal regions were dissected for RNA extraction, library construction, sequencing, and gene expression analysis by a commercial service provider (Novogene Co. Ltd, Beijing, China). Differentially expressed genes (DEGs) were identified and subjected to GO enrichment analysis using clusterProfiler R package. The protein-protein interaction (PPI) network of the DEGs was analyzed via STRING database (https://string-db.org). The RNA-seq data were deposited in the Sequence Read Archive (SRA) repository at NCBI under the accession number PRJNA1134670.

### Morphological Experiments

#### Nissl Staining

Experimental mice underwent anesthesia and transcardial perfusion with PBS, followed by 4% paraformaldehyde (PFA) for fixation. Brains were extracted, fixed in 4% PFA for 24 h, and then cryoprotected in 30% sucrose. Coronal sections of 20 μm thickness were obtained and air-dried. Nissl staining was performed using a staining kit (#G1430, Solarbio) for 1 h. Differentiation in 1% HCl alcohol for 100 s preceded dehydration through graded ethanol series (75%, 85%, 95%, 100% I & II), each for 1 min. Cleared in xylene, sections were mounted with neutral balsam (#G8590, Solarbio).

#### Golgi staining

Mouse brain tissue was resected and immersed in a combination of Solutions A and B from a Golgi staining kit (#PK401, Beijing Boleide, China) at room temperature (RT) in the dark for 14 days. Afterward, tissues were soaked in solution C for 3 days. Sections of 100 μm thickness were cut and re-exposed to solution C for another 3 days, followed by rinsing in distilled water. The slices were then treated with a mixture of Solutions D, E, and double distilled water for 30 min. Dehydration was achieved through graded ethanol series (50%, 75%, 95%, 100% for 4 min each), clearing with xylene, and mounting with a cover slip. High-resolution digital slide scanning (DM6B; Leica, Wetzlar, Germany) was employed to capture images, which were analyzed for dendrite total length and dendritic spine density in hippocampal regions using Sholl analysis.

### Field recordings

#### Preparation of brain slices

21-day-old experimental mice were euthanized to resect brain tissues that were immersed in ice-cold Artificial Cerebrospinal Fluid (ACSF) aerated with a gas mixture of 95% O_2_ and 5% CO_2_. The brain tissues were sliced into 400 μm thick coronal sections using a vibratome (VT1000S; Leica). In current-clamp mode, the recording electrode (3–5 MΩ) filled with 124 mM NaCl was positioned in the stratum radiatum of the hippocampal CA1 area under an optical microscope (EPC-10; HEKA Elektronik Dr. Schulze GmbH, Germany). The stimulation electrode was placed on the Schaffer collateral pathway from CA3 to CA1.

#### Input/Output (I/O) curve recording

The slope, amplitude, and area of the excitatory postsynaptic potentials (EPSPs) in CA1 pyramidal neurons were recorded to generate the input/output curve after receiving a stimuli with increasing intensity (0.02–0.06 mA).

#### Paired-Pulse Facilitation (PPF) recording

The stimulation electrode was set to conduct approximately 50% of the maximum field potential response. EPSPs were elicited using stimulus intervals of 20 ms, 25 ms, 50 ms, 100 ms, and 200 ms. Each cycle contained 10 paired stimuli. The ratio EPSP (2^nd^ s)/EPSP (1st s) in the paired stimuli was analyzed.

#### Long-Term Potentiation (LTP) recording

The stimulation electrode was set to conduct approximately 50% of the maximum field potential response, with a 1 min interval between each sweep. The stable baseline was recorded for 20 min. Long-term potentiation (LTP) was induced by applying theta-burst stimulation (TBS), which consisted of four 10 s-interval episodes, each containing five 5 Hz bursts, with each burst comprising five 100 Hz pulses. Following TBS, LTP was recorded for 40 min using the same baseline method. The magnitude of LTP was assessed by calculating the average slope of 6 EPSPs per minute and comparing the fold change before and after TBS induction.

### Immunohistochemistry

Brain slices were washed with PBS and treated with 3% H_2_O_2_ to inactivate endogenous peroxidase. Antigen retrieval in citrate buffer (0.01 mol/L, pH 6.0) preceded blocking with 10% goat serum at 37 °C for 30 min. Overnight incubation with anti-AEG-1 antibody at 4 °C was followed by PBS washes and incubation with enzyme-labeled goat anti-rabbit IgG (PV-6001; 1:500; ZSGB-Bio, Beijing, China) at 37 °C for 20 min. DAB staining was performed until a brown color developed, followed by hematoxylin nuclear staining. Dehydration through graded ethanol (75%, 80%, 85%, 90%, and 95%) and two washes in 100% ethanol preceded xylene clearing. Sections were mounted with neutral balsam and visualized under a light microscope (DM6; Leica).

### Immunofluorescence

Brain slices were washed with PBS, air-dried at RT and subjected to antigen retrieval by incubating in 0.01 M citrate buffer (pH 6.0) in a microwave for 20 min. After blocking by 1% bovine serum albumin (BSA) at RT for 1 h, sections were incubated with primary antibodies overnight at 4 °C, followed by incubation with appropriate secondary antibodies at RT for 2 h. The nucleus was stained by DAPI-containing mounting medium and imaged under a high-resolution fluorescence microscope. The antibodies used in immunofluorescence were listed in Table S4.

### Western Blot

Tissue samples (hippocampus, neocortex, kidney) were homogenized in ice-cold lysis buffer containing protease inhibitors and phosphatase inhibitors from a total protein extraction kit (KGP250; Nanjing KeyGen Biotech Co., Ltd., China). Lysates were centrifuged at 12,500 g for 10 min at 4 °C. Proteins were separated by SDS-PAGE, transferred to nitrocellulose membranes, blocked with 2% BSA, and incubated with primary antibodies overnight at 4 °C. After washing, membranes were incubated with secondary antibodies for 2 h at RT in the dark. Membranes were imaged using an Odyssey CLx system (#9141-00; LI-COR Biosciences, Lincoln, NE, USA). All original, unprocessed western blot images utilized in this study have been included in the Supplementary Data file. Antibodies used are listed in Table S4.

### Statistical Analysis

Experimental data are presented as mean ± standard deviation (x ± s), with a minimum sample size of three per group (*n* ≥ 3). Tukey’s test compared differences between two groups, one-way ANOVA assessed the effect of a single factor across multiple groups, and two-way ANOVA evaluated the combined effects of two independent variables. Post-hoc tests followed ANOVA for significant differences. Nonparametric tests were applied for non-normal data. In this study, the investigator was blind to the group assignment during the experiment and when evaluating the results. ImageJ, OriginPro, and GraphPrism were used for statistical analysis and data visualization. *P* < *0.05* was considered statistically significant.

## Results

### AEG-1 is highly expressed in normal hippocampal neurons

**Fig. 1:**
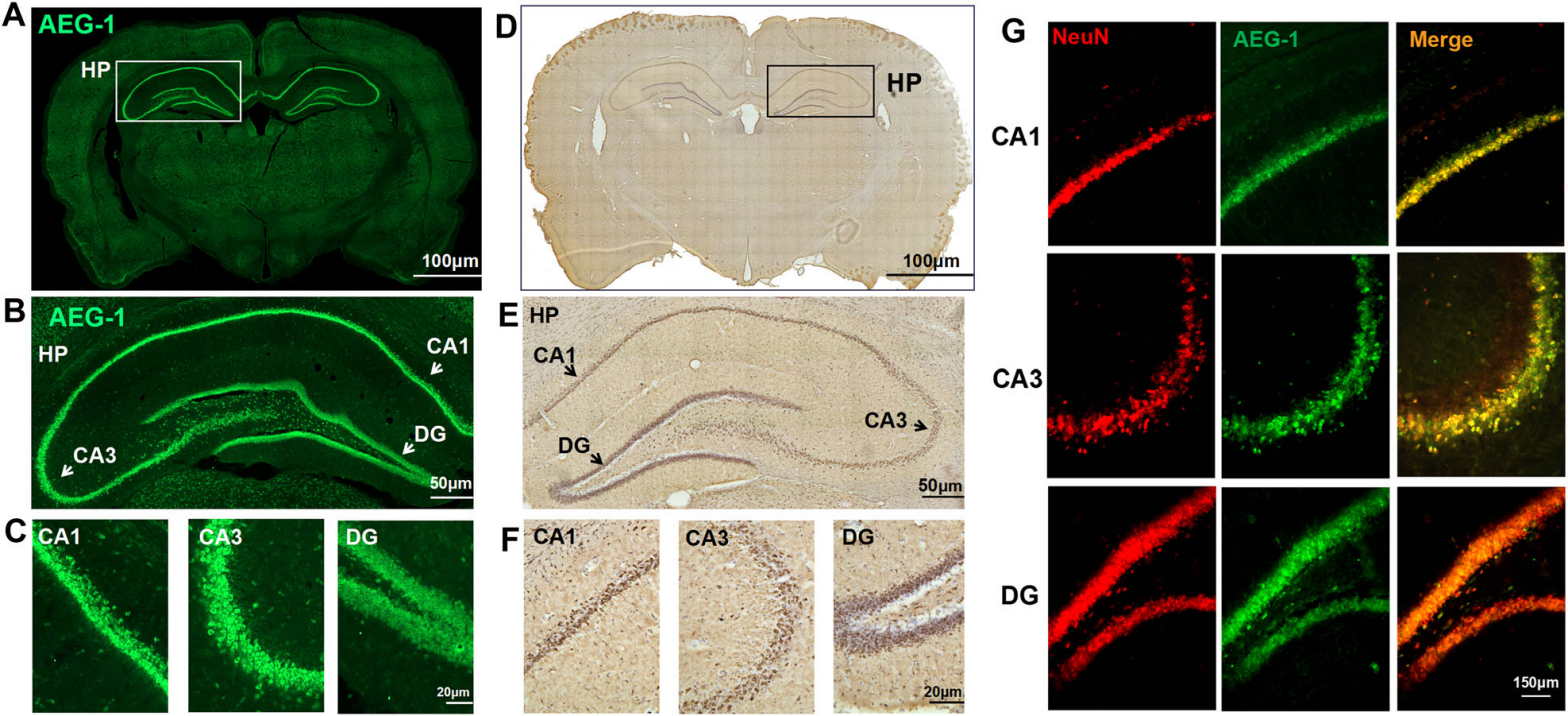
AEG-1 is highly expressed in normal hippocampal neurons. **A** Immunofluorescence staining of AEG-1 for the whole brain slice (scale bar: 100 μm). **B** The entire hippocampal region with AEG-1 staining was selected from (**A**) (scale bar: 50 μm). **C** The AEG-1 staining in CA1, CA3, and DG areas was selected and zoomed in from the hippocampus region in (**B**) (scale bar: 20 μm). **D** Immunohistochemical detection of AEG-1 (brown) in the whole brain slice (scale bar: 100 μm). **E** The hippocampal region was selected and enlarged from (**D**) (scale bar: 50 μm). **F** The CA1, CA3, and DG areas of the hippocampus were selected and enlarged from (**E**) (scale bar: 20 μm). **G** Immunofluorescence detection of AEG-1 in neurons from the CA1, CA3, and DG regions of hippocampus (scale bar: 150 μm).

To explore the physiological role of AEG-1 in brain, we initially determined the expression and distribution of AEG-1 in brain slices. AEG-1 was highly expressed in the pyramidal cell layer of the hippocampus and the DG region in dentate gyrus (Fig. 1A–F). Furthermore, AEG-1 was commonly expressed in hippocampal neurons, astrocytes, and microglia. Interestingly, neurons displayed a higher level of AEG-1 compared to glial cells (Fig. 1G and Fig. S2). The above results suggested that AEG-1 is preferably expressed in normal hippocampal neurons.

### Generation of hippocampus- and neocortex-specific *Aeg-1* knockout (*Aeg-1*^fl/fl^Cre^+^) mice

**Fig. 2:**
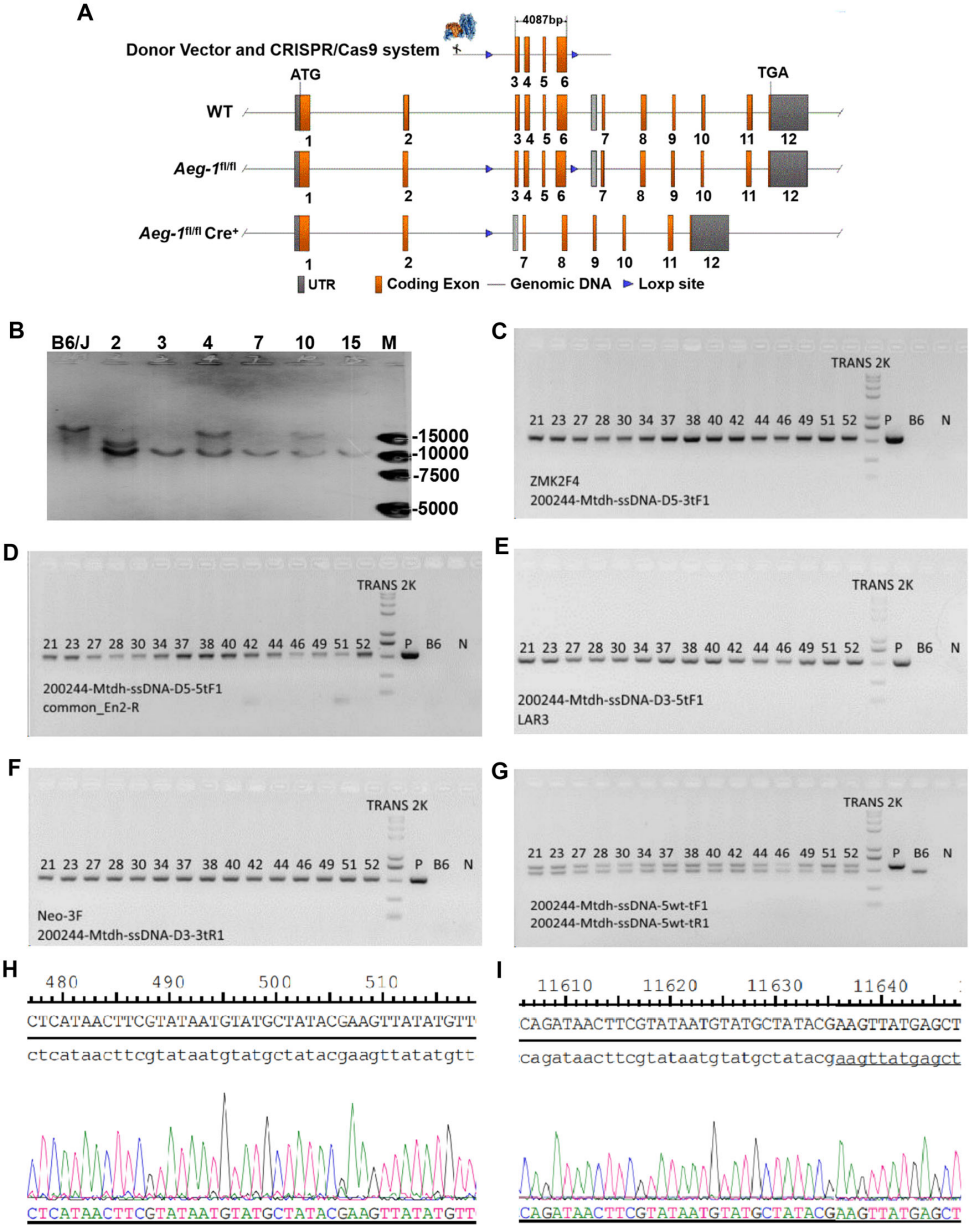
Generation of heterozygous *Aeg-1*^fl/wt^ mice with inserted Loxp sites. **A** Knockout strategy for generating *Aeg-1*^fl/fl^Cre^+^ mice using CRISPR/Cas9 technology and Loxp/Cre system. **B** Southern Blot analysis to detect targeted mouse *Aeg-1* gene locus and copy number. B6/J: WT C57BL/6 J; 2, 3, 4, 7, 10, 15: *Aeg-1*^fl/wt^ positive F0 generation mice; M: DL15,000 Marker bands: 15000 bp\10000 bp\7500 bp\5000 bp. **C**–**G** PCR identification of sites in *Aeg-1*^fl/wt^ F1 and progeny mice: **C** PCR identification of 5′ ssDNA loxP and 3′ homologous arm linker primer site (D5-3) (Fl = 534 bp Wt = none); **D** PCR identification of 5′ ssDNA loxP and 3′ homologous arm linker site (D5-5) (Fl = 527 bp Wt = none); **E** PCR identification of 5′ ssDNA loxP and 3′ homologous arm linker site (D3-5) (Fl = 514 bp Wt = none); **F** PCR identification of 5′ ssDNA loxP and 3′ homologous arm linker site (D3-3) (Fl = 499 bp Wt = none); **G** PCR identification of 5′ screening site (Fl/fl = 570 bp, Fl/wt = 570 bp/471 bp, Wt/wt = 471 bp). B6: negative control, C57BL/6 J mice genomic DNA; N: blank control; P: plasmid positive control; TRANS 2 K PLUS II bands: 8000 bp\5000 bp\3000 bp\2000 bp\1000 bp\750 bp\500 bp \250 bp\100 bp. 21, 23, 27, 28, 30, 34, 37, 38, 40, 42, 44, 46, 49, 51, 52: *Aeg-1*^fl/wt^ positive F1 generation mice. **H**, **I** Sequencing identification of the generated positive F1 mice.

**Fig. 3:**
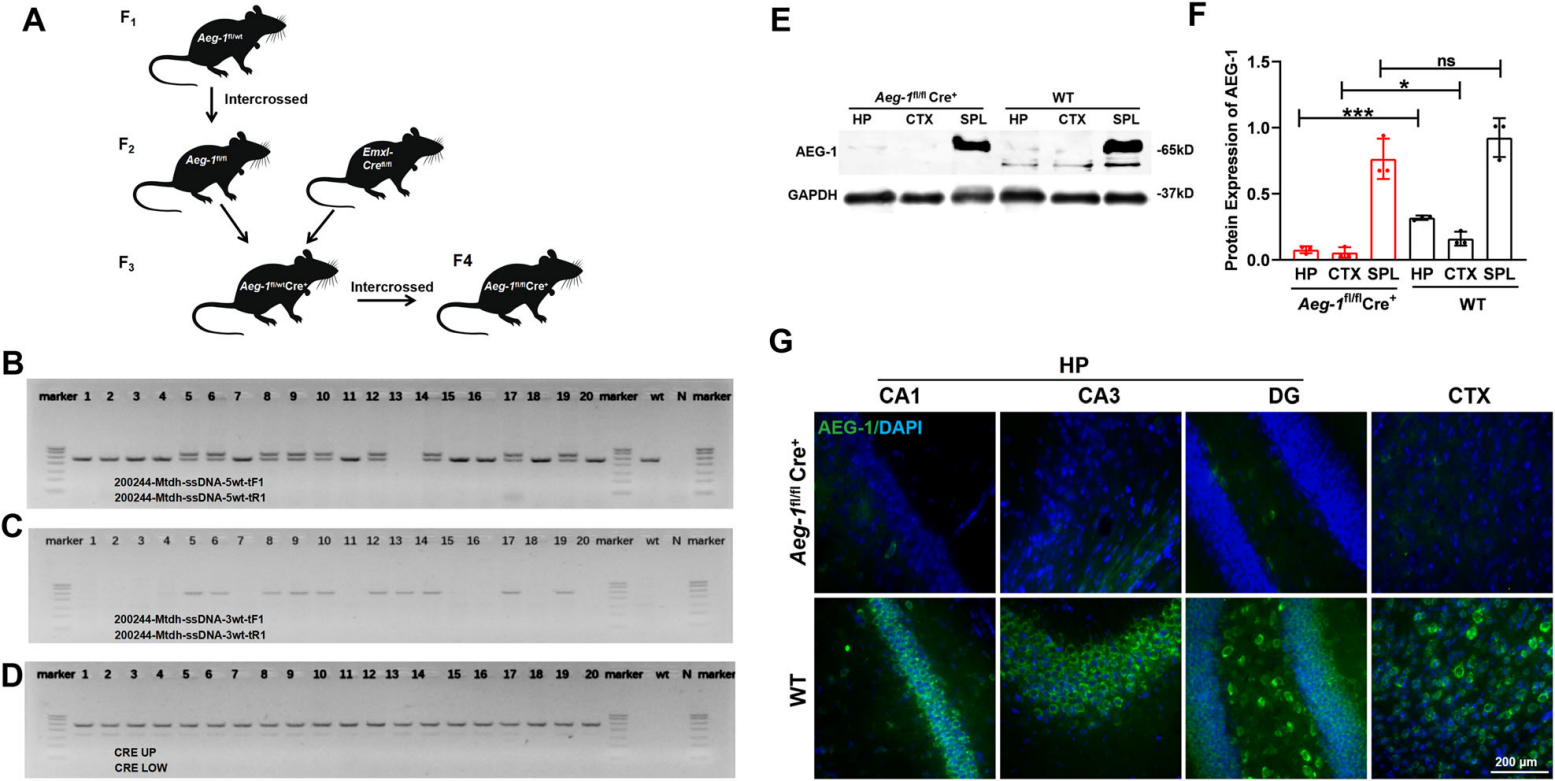
Successful breeding of *Aeg-1*^fl/fl^Cre^+^ mice. **A** Breeding strategy of *Aeg-1*^fl/fl^Cre^+^ mice. **B**–**D** PCR identification of sites in *Aeg-1*^fl/fl^Cre^+^ mice: (**B**) PCR identification of 5′ screening site (Fl/fl = 570 bp, Fl/wt = 570 bp/471 bp, Wt = 471 bp); (**C**) PCR identification of D5-5 site (Fl = 527 bp, Wt = none); (**D**) PCR identification results of Cre site (T = 481 bp); 1-20: *Aeg-1*^fl/wt^Cre^+^ mice number; wt: WT mice; N: blank control; Marker bands: 700 bp/600 bp/500 bp/400 bp/300 bp/200 bp/100 bp. **E**, **F** Western blot results of AEG-1 protein expression in *Aeg-1*^fl/fl^Cre^+^ mice and WT mice: **E** Western blotting analysis of AEG-1 and the quantification was presented in (**F**). HP brain hippocampal tissue, CTX brain cortical tissue, SPL kidney tissue. Results analyzed by Tukey’s test. *p < 0.05, ***p < 0.001, ns*:* no significant. **G** Immunofluorescence detection of *AEG-1* protein expression in the hippocampus and cortex of *Aeg-1*^fl/fl^Cre^+^ mice and WT mice. Scale Bar: 200 μm.

Since high AEG-1 expression was confirmed in normal hippocampal neurons, next we generated mouse models rendered hippocampus- and neocortex-specific *Aeg-1* knockout to demonstrate the physiological function of AEG-1 (Fig. 2A). The *Aeg-1*^fl/wt^ F0 positive mice were validated by the correct targeted band at 10,000 bp (Fig. 2B). These mice were subjected to self-crossing to obtain the heterozygous *Aeg-1*^fl/wt^ mice with loxP site insertion, which was validated by electrophoresis following PCR at five sites (Fig. 2C–G) and by Sanger sequencing (Fig. 2H, I). Subsequently, *Aeg-1* floxP homozygous and Cre-positive (*Aeg-1*^fl/fl^Cre^+^) mice were obtained by breeding (Fig. 3A) and the specific *Aeg-1* knockout in *Aeg-1*^fl/fl^Cre^+^ mice was validated by PCR at three sites (Fig. 3B–D). In addition, the expression of AEG-1 protein was largely abrogated in the hippocampus and cortex of *Aeg-1*^fl/fl^Cre^+^ mice (Fig. 3E–G), which stood for the successful generation of *Aeg-1* knock out mice.

### *Aeg-1*-deficiency is associated with impaired learning and memory abilities and depressive-like behaviors

**Fig. 4:**
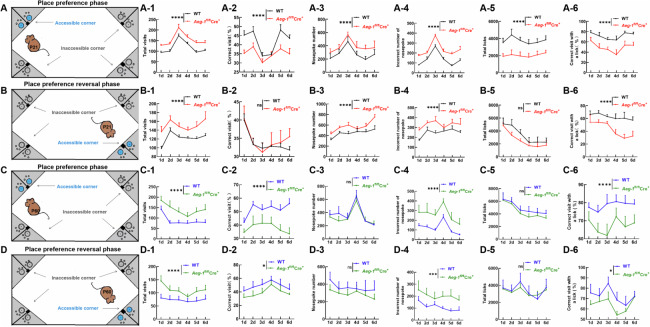
*Aeg-1*-deficiency is associated with impaired learning and memory abilities. **A** Place preference and (**B**) place preference reversal paradigm for juvenile mice; (A-1, B-1) Quantitative analysis of the total number of visits to the four corners (*Aeg-1*^fl/fl^Cre^*+*^ juvenile mice and WT juvenile mice); (A-2, B-2) Quantitative analysis of the percentage of visits to the correct corner (*Aeg-1*^fl/fl^Cre^+^ juvenile mice and WT juvenile mice); (A-3, B-3) Quantitative analysis of the total number of nose pokes (*Aeg-1*^fl/fl^Cre^+^ juvenile mice and WT juvenile mice); (A-4, B-4) Quantitative analysis of the number of nose pokes in the incorrect corners (*Aeg-1*^fl/fl^Cre^+^ juvenile mice and WT juvenile mice); (A-5, B-5) Quantitative analysis of the total number of drinking events (*Aeg-1*^fl/fl^Cre^+^ juvenile mice and WT juvenile mice); (A-6, B-6) Quantitative analysis of the percentage of successful visits to the correct corner with at least one lick of water, out of the total number of visits to the correct corner (*Aeg-1*^fl/fl^Cre^*+*^ juvenile mice and WT juvenile mice). **C** Place preference and (**D**) place preference reversal paradigm for adult mice; (C-1, D-1) Quantitative analysis of the total number of visits to the four corners (*Aeg-1*^fl/fl^Cre^+^ adult mice and WT adult mice); (C-2, D-2) Quantitative analysis of the percentage of visits to the correct corner (*Aeg-1*^fl/fl^Cre^+^ adult mice and WT adult mice); (C-3, D-3) Quantitative analysis of the total number of nose pokes (*Aeg-1*^fl/fl^Cre^+^ adult mice and WT adult mice); (C-4, D-4) Quantitative analysis of the number of nose pokes in the incorrect corners (*Aeg-1*^fl/fl^Cre^+^ adult mice and WT adult mice); (C-5, D-5) Quantitative analysis of the total number of drinking events (*Aeg-1*^fl/fl^Cre^+^ adult mice and WT adult mice); (C-6, D-6) Quantitative analysis of the percentage of successful visits to the correct corner with at least one lick of water, out of the total number of visits to the correct corner (*Aeg-1*^fl/fl^Cre^+^ adult mice and WT adult mice). (Results analyzed by two-way ANOVA. *n* = 15 per group, ^ns^*P* > 0.05, ^*^P < 0.05, ^***^P < 0.001, ^****^P < 0.0001).

**Fig. 5:**
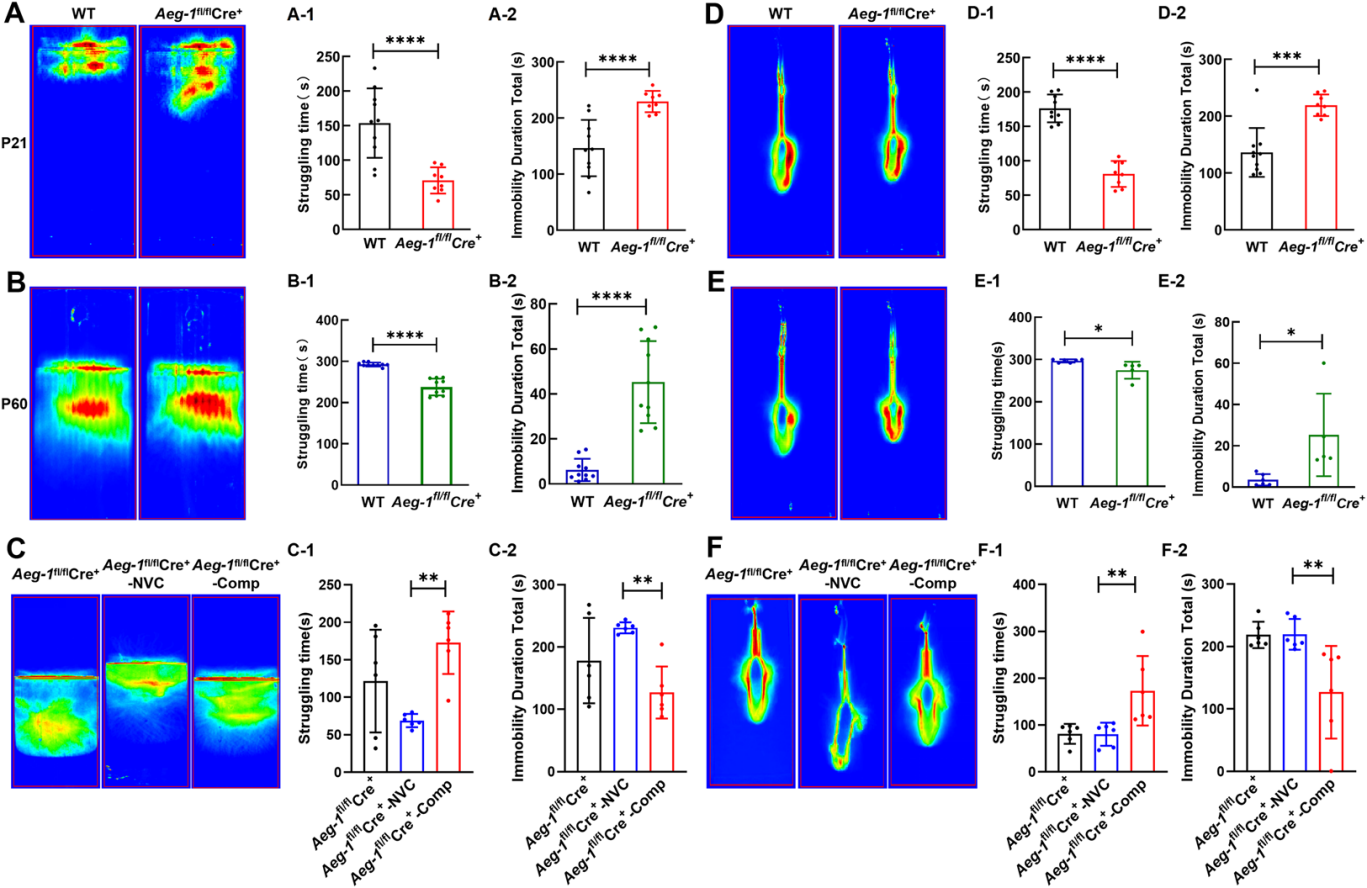
*Aeg-1*^fl/fl^Cre^+^ juvenile and adult mice exhibit depressive-like behaviors. **A** Heatmap of the swimming trajectory of *Aeg-1*^fl/fl^Cre^+^ juvenile mice and WT juvenile mice in the forced swimming test. Quantitative analysis of (A-1) the struggling time and (A-2) the total immobility duration in the forced swimming test, compared *Aeg-1*^fl/fl^Cre^+^ juvenile mice (*n* = 8) to WT juvenile mice (*n* = 10). **B** Heatmap of the swimming trajectory of *Aeg-1*^fl/fl^Cre^+^ adult mice and WT adult mice in the forced swimming test. Quantitative analysis of (B-1) the struggling time and (B-2) the total immobility duration in the forced swimming test, compared *Aeg-1*^fl/fl^Cre^+^ adult mice to WT adult mice (*n* = 10 per group). **C** Heatmap of the swimming trajectory of *Aeg-1*^fl/fl^Cre^+^ mice, *Aeg-1*^fl/fl^Cre^+^-NVC mice and *Aeg-1*^fl/fl^Cre^+^-Comp mice in the forced swimming test. Quantitative analysis of (C-1) the struggling time and (C-2) the total immobility duration in the forced swimming test, compared *Aeg-1*^fl/fl^Cre^+^ mice, *Aeg-1*^fl/fl^Cre^+^-NVC mice and *Aeg-1*^fl/fl^Cre^+^-Comp mice (*n* = 6 per group). **D** Heatmap of *Aeg-1*^fl/fl^Cre^+^ juvenile mice and WT mice trajectory in the tail suspension test. Quantitative analysis of (D-1) the struggling time and (D-2) the total immobility duration in the tail suspension test, compared *Aeg-1*^fl/fl^Cre^+^ juvenile mice to WT juvenile mice (*n* ≥ 8 per group). **E** Heatmap of *Aeg-1*^fl/fl^Cre^+^ adult mice and WT adult mice trajectory in the tail suspension test. Quantitative analysis of the (E-1) struggling time and (E-2) the total immobility duration in the tail suspension test, compared *Aeg-1*^fl/fl^Cre^+^ adult mice (*n* = 5) to WT adult mice (*n* = 6). **F** Heatmap of *Aeg-1*^fl/fl^Cre^+^ mice, *Aeg-1*^fl/fl^Cre^+^-NVC mice and *Aeg-1*^fl/fl^Cre^+^-Comp mice trajectory in the tail suspension test. Quantitative analysis of the (F-1) struggling time and (F-2) the total immobility duration in the tail suspension test, compared *Aeg-1*^fl/fl^Cre^+^ mice, *Aeg-1*^fl/fl^Cre^+^-NVC mice and *Aeg-1*^fl/fl^Cre^+^-Comp mice (*n* = 6 per group).(Results from (**A**, **B**, **D**, **E**) analyzed by Tukey’s test, results from (**C**, **F**) analyzed by one-way ANOVA. Juvenile: 21-day-old, adult: 60-day-old. ^*^P < 0.05, ^***^P < 0.001^, ****^P < 0.0001).

**Fig. 6:**
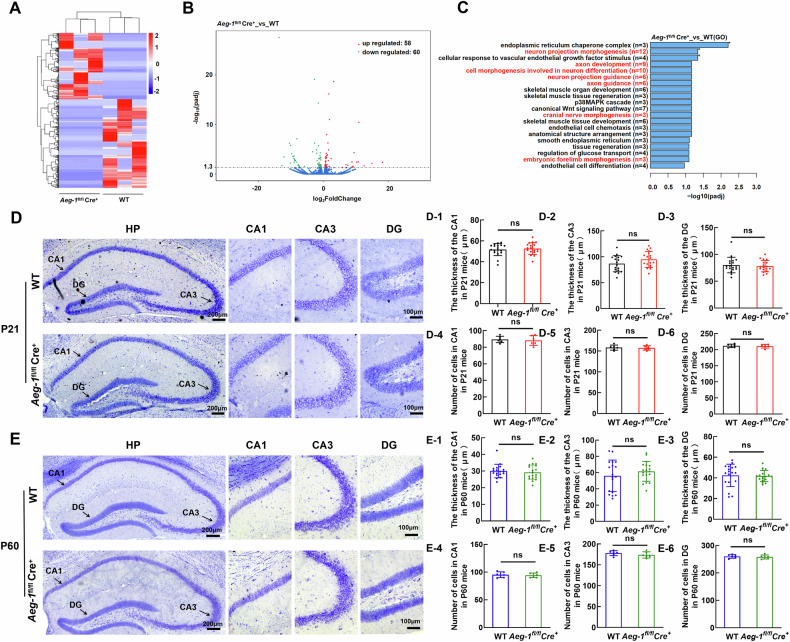
*Aeg-1* deficiency leads to abnormal hippocampal neuronal morphology without altering hippocampal thickness or cell numbers. **A**–**C** GO enrichment analysis on DEGs (*n* = 3 per group, DEGs criteria: |Fold change | > 2 and adjusted p-value < 0.05): **A** Hierarchical clustering heatmap of DEGs (Red: upregulated genes; Blue: downregulated genes, a darker color indicates a higher expression level). **B** Volcano plot of DEGs (Red: upregulated genes; Green: downregulated genes). **C** Top 20 pathways and biological processes were presented from the enrichment analysis of DEGs in GO annotation. **D**, **E** Thickness and cell number of different regions in the hippocampus of *Aeg-1*^fl/fl^Cre^+^ mice and WT mice: **D** Nissl staining of the hippocampus in *Aeg-1*^fl/fl^Cre^+^ juvenile mice and WT juvenile mice (scale bar: 200 μm). The regions of CA1, CA3 and DG were separately presented (scale bar:100 μm). D1–D3 Quantitative analysis of the hippocampal CA1, CA3, and DG region thickness and D4–D6 cell numbers in *Aeg-1*^fl/fl^Cre^+^ juvenile mice and WT juvenile mice. **E** Nissl staining of the hippocampus in *Aeg-1*^fl/fl^Cre^+^ adult mice and WT adult mice (scale bar: 200 μm). The regions of CA1, CA3 and DG were separately presented (scale bar:100 μm). E1-E3 Quantitative analysis of the hippocampal CA1, CA3, and DG region thickness and E4-E6 cell numbers in adult *Aeg-1*^fl/fl^Cre^+^ and WT mice. (Results analyzed by Tukey’s test. Juvenile: 21-day-old, adult: 60-day-old. *n* = 6 per group, ^ns^*P* > 0.05).

Interestingly, hippocampus- and neocortex-specific *Aeg-1* deficiency was irrelevant to the survival rate but caused weight loss in *Aeg-1*^fl/fl^Cre^+^ mice (Fig. S3). Next, we determined the effect of *Aeg-1* deletion on the behavior of juvenile and adult mice. *Aeg-1*^fl/fl^Cre^+^ juvenile mice displayed more total number of visits and nose-pokes in place learning phase and reversal place learning phase (Fig. 4A-1, A-3, B-1, B-3). Conversely, a dropped frequency of visits to the correct corners was observed in *Aeg-1*^fl/fl^Cre^+^ juvenile mice (Fig. 4A-6, B-6). Similar results were obtained in *Aeg-1*-deficient adult mice (Fig. 4C-1, D-1, C-6, D-6). Therefore, *Aeg-1* deficiency was associated with the impaired learning and memory abilities in juvenile and adult mice.

In addition, we assessed the depressive-like behaviors of *Aeg-1*^fl/fl^Cre^+^ juvenile and adult mice by a forced swimming test and a tail suspension test. *Aeg-1*^fl/fl^Cre^+^ juvenile mice exhibited a shortened struggling time (Fig. 5A-1, D-1) and a prolonged immobility time compared to WT juvenile mice (Fig. 5A-2, D-2). Similar results can be also observed in *Aeg-1*^fl/fl^Cre^+^ adult mice (Fig. 5B-1, B-2, E-1, E-2). To further substantiate the causal relationship between *Aeg-1* deficiency and these behavioral deficits, we conducted a rescue experiment by using stereotaxic injection to deliver an adeno-associated virus (AAV) overexpressing AEG-1 to re-express AEG-1 specifically in the hippocampus and neocortex of *Aeg-1*^fl/fl^Cre^+^ mice (Fig. S4). Re-expression of AEG-1 in the *Aeg-1*^fl/fl^Cre^+^ mice resulted in an increase in struggling time and a decrease in immobility time in both the forced swimming test and the tail suspension test (Fig. 5C-1, C-2, F-1, F-2). Therefore, *Aeg-1* deficiency was associated with depressive-like behaviors in juvenile and adult mice, and these defects were rescued by specific re-expression of *Aeg-1* in the hippocampus and neocortex.

### *Aeg-1* deficiency causes abnormal morphology of hippocampal neurons

**Fig. 7:**
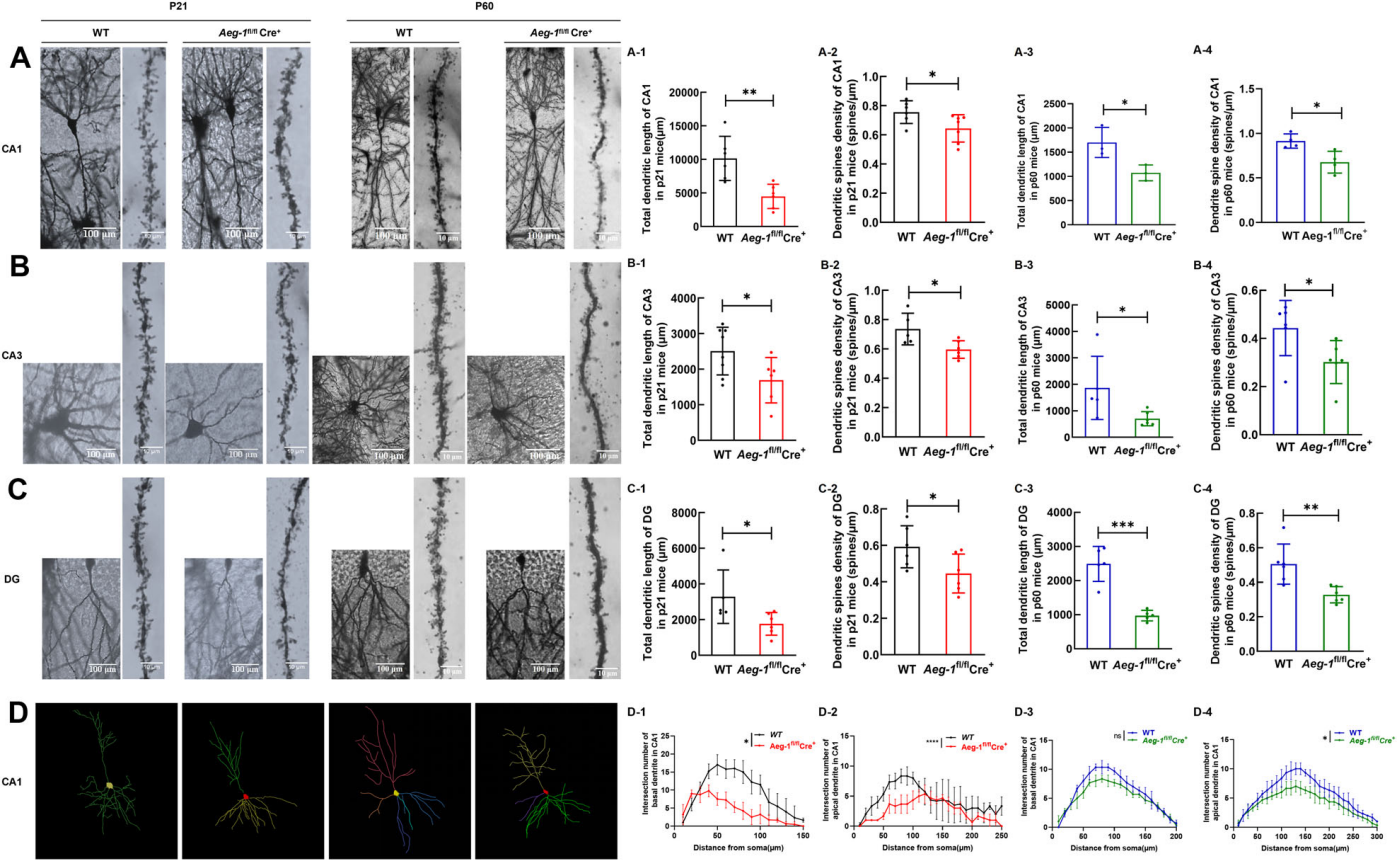
Dendritic length and dendritic spine density of neurons in different hippocampal regions of *Aeg-1*^fl/fl^Cre^+^ mice and WT mice. **A** Golgi staining of neuronal dendrites and apical terminal dendritic spines in the CA1 region of the hippocampus derived from *Aeg-1*^fl/fl^Cre^+^ mice and WT mice. **B** Golgi staining of neuronal dendrites and apical terminal dendritic spines in the CA3 region of the hippocampus derived from *Aeg-1*^fl/fl^Cre^+^ mice and WT mice. **C** Golgi staining of neuronal dendrites and apical terminal dendritic spines in the DG region of the hippocampus derived from *Aeg-1*^fl/fl^Cre^+^ mice and WT mice. Quantitative analysis of (A1, B1, C1) dendritic length and (A2, B2, C2) dendritic spine density of hippocampal neurons in CA1, CA3, and DG regions of *Aeg-1*^fl/fl^Cre^+^ juvenile mice and WT juvenile mice. Quantitative analysis of (A3, B3, C3) dendritic length and (A4, B4, C4) dendritic spine density of hippocampal neurons in CA1, CA3, and DG regions of *Aeg-1*^fl/fl^Cre^+^ adult mice and WT adult mice. **D** Camera lucida drawing of neuronal dendrites in the CA1 region of the hippocampus derived from *Aeg-1*^fl/fl^Cre^+^ mice and WT mice; (D1, D2) The complexity of basal and apical dendrites in CA1 neurons of the hippocampus derived from *Aeg-1*^fl/fl^Cre^+^ juvenile mice and WT juvenile mice. (D3, D4) The complexity of basal and apical dendrites in CA1 neurons of the hippocampus derived from *Aeg-1*^fl/fl^Cre^+^ adult mice and WT adult mice.(Results analyzed by Tukey’s test. Juvenile: 21-day-old, adult: 60-day-old. *n* = 3 per group, *P < 0.05, **P < 0.01; Golgi staining of neurons, scale bar: 100 μm; Golgi staining of dendritic spines, scale bar: 10 μm).

To elucidate the mechanism underlying the impaired learning and memory abilities caused by *Aeg-1* deletion in mice, we performed RNA-Seq analysis and screened differentially expressed genes (DEGs) in the hippocampus of *Aeg-1*^fl/fl^Cre^+^ mice. A total of 58 up-regulated genes and 60 down-regulated genes were obtained respectively in *Aeg-1*-deficient hippocampus compared to *Aeg-1*-proficient hippocampus (Fig. 6A, B). The DEGs were mainly enriched in the gene sets mediating hippocampal neuron morphology (Fig. 6C), which suggested that *Aeg-1* may regulate neuronal morphogenesis. *Aeg-1* status was dispensable for the thickness changes and cell number changes of hippocampal CA1, CA3, and DG regions of juvenile and adult mice (Fig. 6D1–D6, E1–E6). Deletion of *Aeg-1* shortened the total dendritic length of hippocampal neurons in CA1, CA3, and DG regions in juvenile mice (Fig. 7A-C, A–1, B-1, C-1). The dendritic spine density also declined following the *Aeg-1* knock out in juvenile mice (Fig. 7A-C, A-2, B-2, C-2). Similar results were observed in *Aeg-1*^fl/fl^Cre^+^ adult mice (Fig. 7A-C, A-3, A-4, B-3, B-4, C-3, C-4). The CA1 region was specifically analyzed due to its critical role in learning, memory, and synaptic plasticity. Consistent with these observations, Sholl analysis showed decreased apical dendrite complexity in both juvenile and adult mice after Aeg-1 knockout, but no difference in basal dendrites of adult mice (Fig. 7D, D1–D4). Thus, it was revealed that AEG-1 affects neuronal dendrite formation and complexity, particularly apical dendrites, within the CA1 region. Therefore, AEG-1 is closely related to the dendrite formation and complexity of neurons in this specific area.

### *Aeg-1* knockout impairs synaptic function in hippocampal neurons

**Fig. 8:**
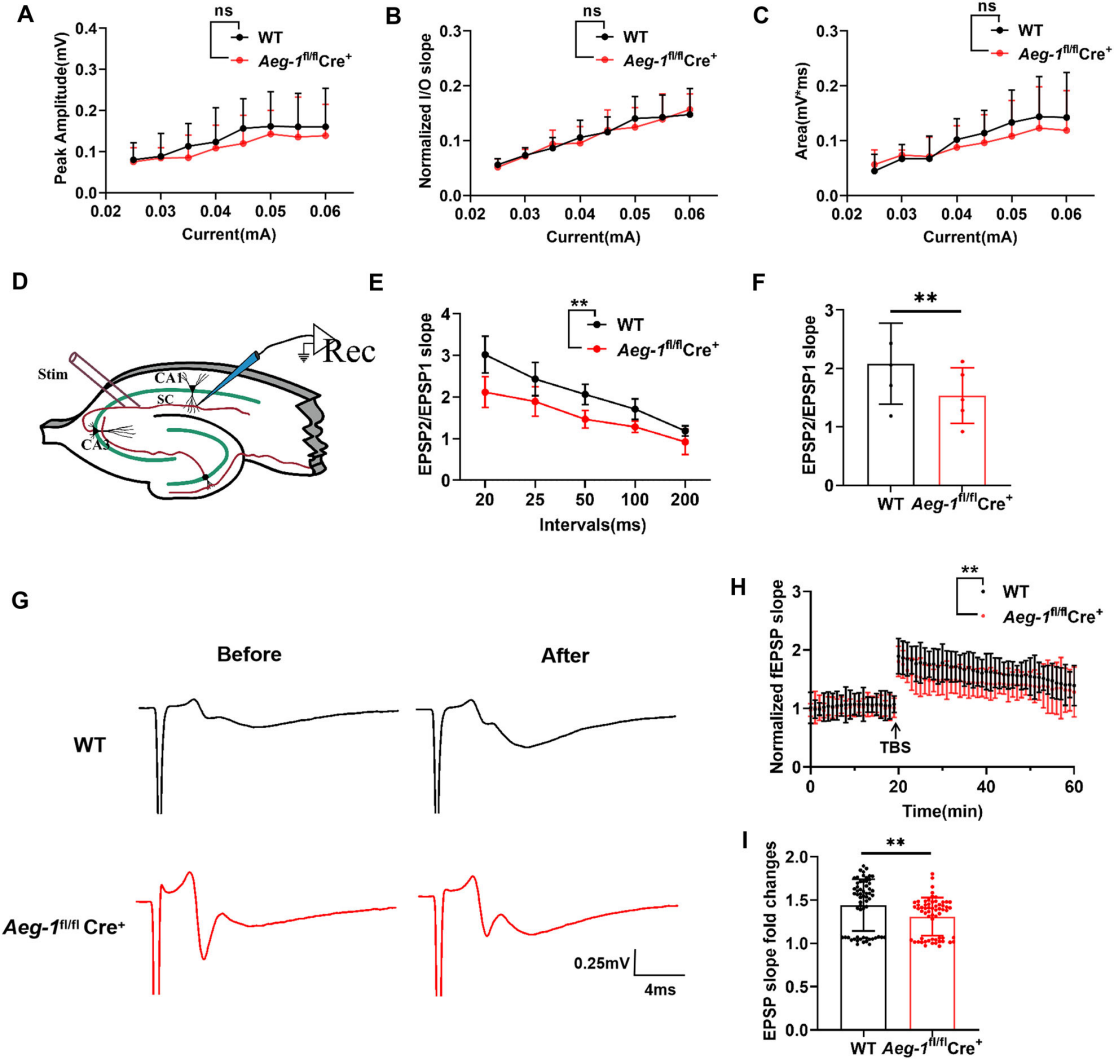
I/O curves, PPF responses, and LTP of field potentials in the CA3-CA1 region of brain slices from *Aeg-1*^fl/fl^Cre^+^ mice and WT mice. **A**–**C** I/O curves of EPSP amplitude (mV), slope (mV/ms), and area (mV*ms) at different stimulation intensities (WT: 14 slices from 7 mice, *Aeg-1*^fl/fl^Cre^+^: 12 slices from 5 mice). **D** Schematic diagram of the field potential stimulation electrode and recording electrode positions in the CA3-CA1 region of mouse brain slices. **E** PPF responses of EPSP2/EPSP1 with stimulation intervals of 20 ms, 25 ms, 50 ms, 100 ms, and 200 ms (WT: 10 slices from 4 mice, *Aeg-1*^fl/fl^Cre^+^: 11 slices from 5 mice, **P < 0.01), along with the total EPSP2/EPSP1 slope in (**F**). **G** Representative recordings of LTP responses in the CA3-CA1 region of mouse brain slices (Scale: 0.25 mV, 4 ms). **H** EPSP slopes and **I** statistical analysis of LTP in the CA3-CA1 region of mouse brain slices in the indicated groups (WT: 11 slices from 6 mice, *Aeg-1*^fl/fl^Cre^+^: 12 slices from 5 mice, **P < 0.01). (Results in (**A**, **B**, **C**, **E**, **H**) analyzed by two-way ANNOVA, and results in (**F**, **I**) analyzed by Tukey’s test).

Next, we asked whether *Aeg-1* is critical for the synaptic function of hippocampal neurons. To this end, we performed field potential recordings in the CA3-CA1 regions of hippocampal brain slices derived from *Aeg-1*^fl/fl^Cre^+^ mice and WT mice. The changes of EPSP amplitude, slope and area were unsignificant in *Aeg-1*^fl/fl^Cre^+^ mice compared to WT mice assessed by input-output (I/O) curves (Fig. 8A–C), indicating that AEG-1 is irrelevant in hippocampal basal synaptic strength and transmission. The paired-pulse facilitation (PPF) response was reduced in the hippocampal CA1 region of *Aeg-1*^fl/fl^Cre^+^ mice compared to WT mice (Fig. 8E, F), suggesting that the loss of *Aeg-1* increases the probability of vesicle releasing at presynaptic axon terminals. The long-term potentiation (LTP) was induced in the hippocampus of both *Aeg-1*^fl/fl^Cre^+^ mice and WT mice, whereas the LTP was induced extensively in *Aeg-1*-proficient hippocampal following TBS stimulation (Fig. 8G–I). The above results implied that *Aeg-1* may mediate the long-term enhancement of excitatory synaptic transmission.

## Discussion

In this study we demonstrated physiological functions of AEG-1 in hippocampus in transgenic mouse models. In line with previous studies [11], AEG-1 tended to be highly expressed in hippocampal pyramidal neurons in physiological conditions. The hippocampus is essential for the formation and retrieval of episodic memories. In addition, the emotional regulation relies on the hippocampus function [12–16]. Patients with reduced hippocampal volume present major depressive disorder [17]. Therefore, high expression of AEG-1 is crucial for maintaining hippocampus functions in episodic memory formation and retrieval, as well as emotional regulation. Indeed, *Aeg-1* deletion impaired the learning and memory abilities. Mice with *Aeg-1* deficiency displayed depressive-like behaviors. Our results pointed out that AEG-1 is required to maintain normal hippocampal function in physiological conditions. Notably, weight loss happened following *Aeg-1* deletion in the model. The weight loss was also observed in a hepatocellular carcinoma (HCC) mouse model with deficient-*Aeg-1* [18]. Inhibition of AEG-1 induces the expression of DIO1, an enzyme that converts L-thyroxine (T4) into 3,5,3′-triiodothyronine (T3). Given the T3’s central role in regulating metabolism, energy expenditure and weight management [19, 20], the elevation of T3 levels following AEG-1 inhibition likely exerts an indirect effect on the metabolic rate of mice, and thereby leads to weight changes.

AEG-1 is associated with astrocyte reactivation and involved in the progression of neurological diseases [5, 21, 22]. Amyotrophic lateral sclerosis (ALS) motor neuron activity is weakened by AEG-1 repression [7]. Additionally, AEG-1 reduces the anti-apoptotic ability of dopamine neurons in Alzheimer’s disease (AD) patients [8]. AEG-1 also contributes to granulosa cell diffusion in a temporal lobe epilepsy model [4]. Therefore, AEG-1 dysregulation confers the onset of neuro-diseases.

Here, the DEGs in *Aeg-1* deficient hippocampal neuron were enriched in the gene sets regulating neuronal morphology. Thereby, AEG-1 is possibly required to maintain neuronal morphology in physiological conditions. Numerous studies have shown that hippocampus structural damages, such as reductions in volume and thickness, as well as abnormal changes in dendritic length and dendritic spine density, have varying detrimental effects on individuals [23–26]. Indeed, loss of *Aeg-1* reduced the length and the spine density of dendrites, which are sufficient to cause structural damage in hippocampus. *Aeg-1* deficiency therefore contributed to cognitive function impairment and depressive-like mood. Since juvenile phase is indispensable for the neuronal maturation [27], AEG-1 may determine the fate of hippocampus at this phase.

Learning and memory abilities are synaptic plasticity-dependent [28]. AEG-1 may therefore affect learning and memory via regulating synaptic plasticity. Interestingly, AEG-1 was not responsible for the basal synaptic strength of the hippocampus. Loss of *Aeg-1* increased the probability of vesicle releasing at presynaptic axon terminals in PPF response, possibly due to the residual Ca^2+^ in the presynaptic terminal from the first pulse [29]. Furthermore, the synaptic transmission efficiency was compromised in mice rendered *Aeg-1* deficiency. Two glutamate receptors, AMPA and NMDA, are activated in the increased LTP [30], therefore, AEG-1 possibly can affect LTP via AMPA and NMDA but the hypothesis requires further experimental evidence. The absence of AEG-1 likely impacts the signaling transmission and enhancement mechanisms between neurons, which subsequently impacts synaptic plasticity and memory function.

To investigate the mechanisms through which *Aeg-1* deficiency alters neuronal morphology and function, we analyzed the downstream signaling pathways influenced by the absence of AEG-1. Notably, our GO analysis indicated a significant impact on the p38 MAPK cascade, a component of the MAPK signaling pathway, which is known to be essential for synaptic plasticity and learning and memory processes [31]. This observation suggests that AEG-1 may be regulating neuronal processes via the MAPK signaling pathway. Furthermore, considering the established role of the PI3K/Akt pathway in cancer research, where it is regulated by AEG-1, and its distinct function in neurons, promoting synaptic plasticity and cognitive processes, we postulate that AEG-1 might also be influencing neuronal morphology and function through the PI3K/Akt pathway [32]. These insights provide deeper understanding into the mechanisms underlying *Aeg-1*’s role in neurons, particularly its impact on synaptic plasticity, learning, memory, and cognitive processes.

In conclusion, specific *Aeg-1* deletion in hippocampal neurons razes neuronal substructure, dendritic structure and synaptic function, which leads to depression and impairment of learning and memory in mice. Our findings not only provide new perspectives and clues for understanding the pathogenesis of cognitive and mood disorders, but also offers novel targets and strategies for the treatment of neurological diseases. However, further studies are warranted to show how AEG-1 mediates the changes in neuronal morphology.

## Supplementary information

Supplementary Information

## Data Availability

The datasets used and/or analyzed during the current study are available from the corresponding author on reasonable request.
