# Supplementary material for: Hippocampus- and neocortex-specific deletion of Aeg-1 causes learning memory impairment and depression in mice
Source: Cell Death Dis. 2025 Mar 23;16(1):199. doi: 10.1038/s41419-025-07508-0 (PMC11930984; doi:10.1038/s41419-025-07508-0)
Supplement: Supplementary file 1 — Supplementary Information [file 41419_2025_7508_MOESM1_ESM.pdf]

Supplementary Information

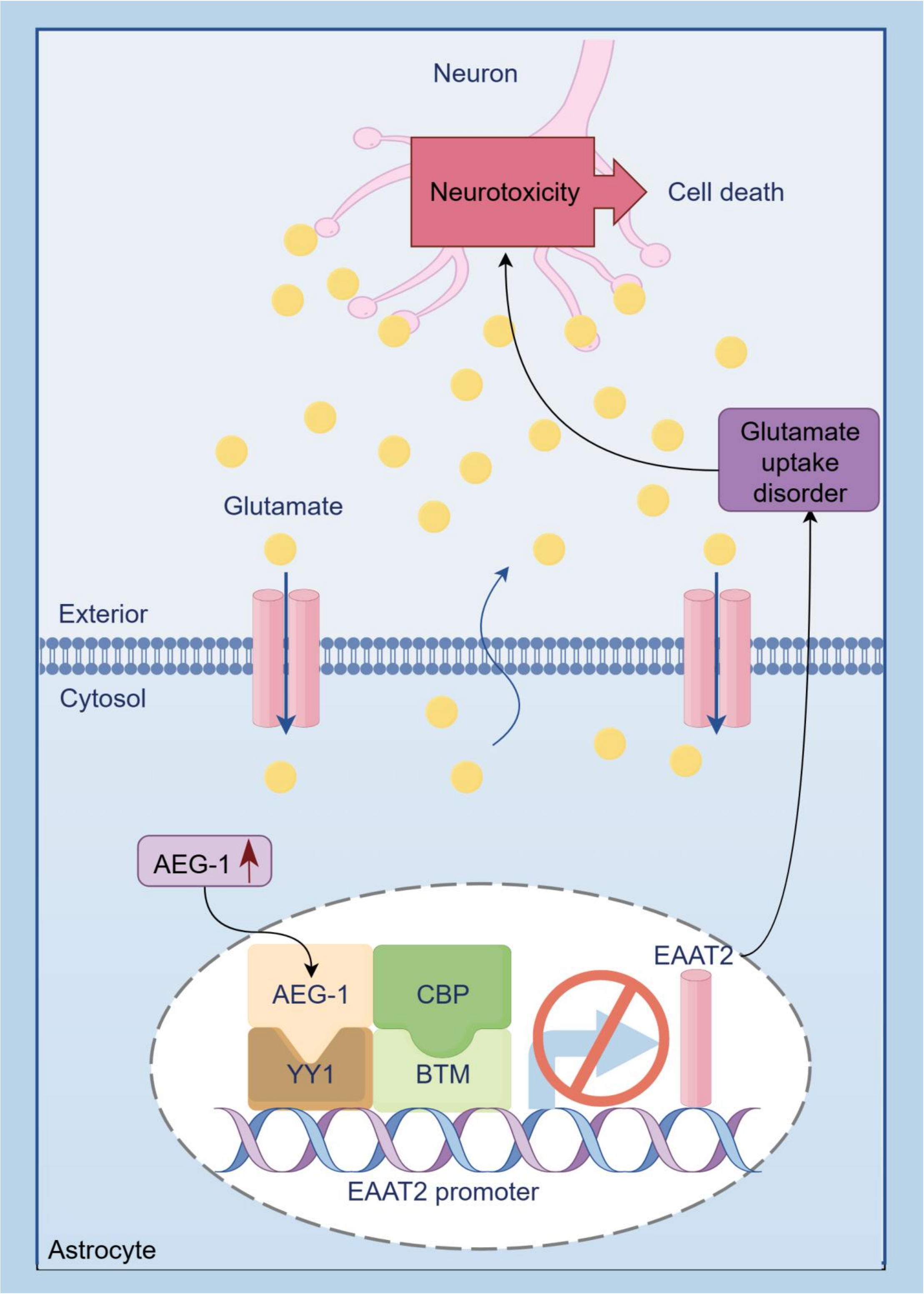

Fig. S1 The regulatory mechanism of AEG-1 in astrocytes during the progression of neurological diseases. Increased AEG-1 negatively regulates the expression of EAAT2, which normally functions as a primary glutamate transporter, thereby inducing glutamate excitotoxicity and neuronal cell death (By Figdraw).

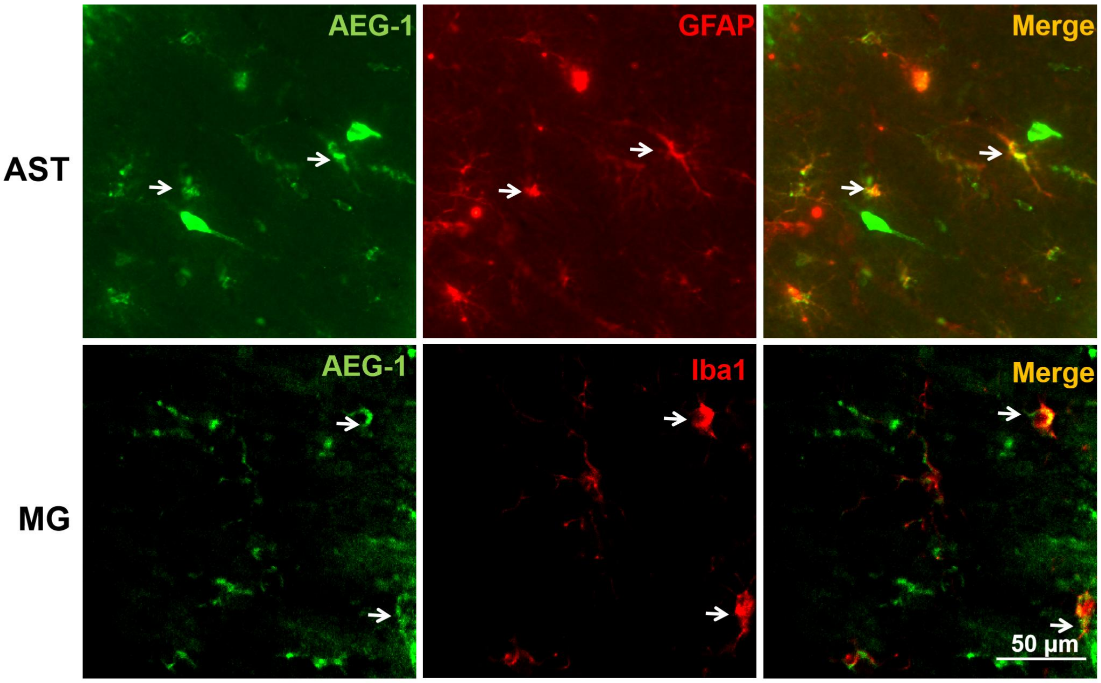

Fig. S2 AEG-1 is expressed in hippocampal neurons, astrocytes, and microglia. Red: GFAP/Iba1; Green: AEG-1; White arrow: the co-localization of AEG-1 with GFAP or Iba1, indicating the expression of AEG-1 in astrocytes (AST, labeled by GFAP) and microglia (MG, labeled by Iba1), scale bar : 50 μm.

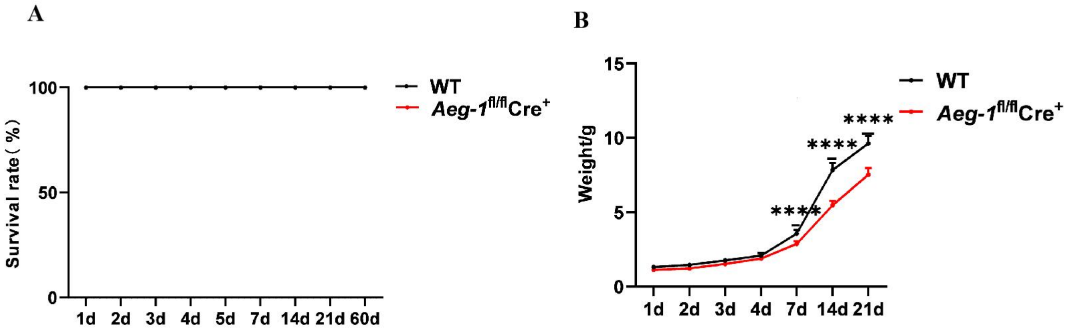

Fig. S3 Survival rate and weight of *Aeg-1*$^{fl/fl}$Cre^{+} mice. (A) Survival rate of *Aeg-1*$^{fl/fl}$Cre^{+} juvenile mice and WT juvenile mice within 60 postnatal days; (B) Weight of *Aeg-1*$^{fl/fl}$Cre^{+} juvenile mice and WT juvenile mice within 21 postnatal days (n = 15 per group, $^{****}P < 0.0001$).

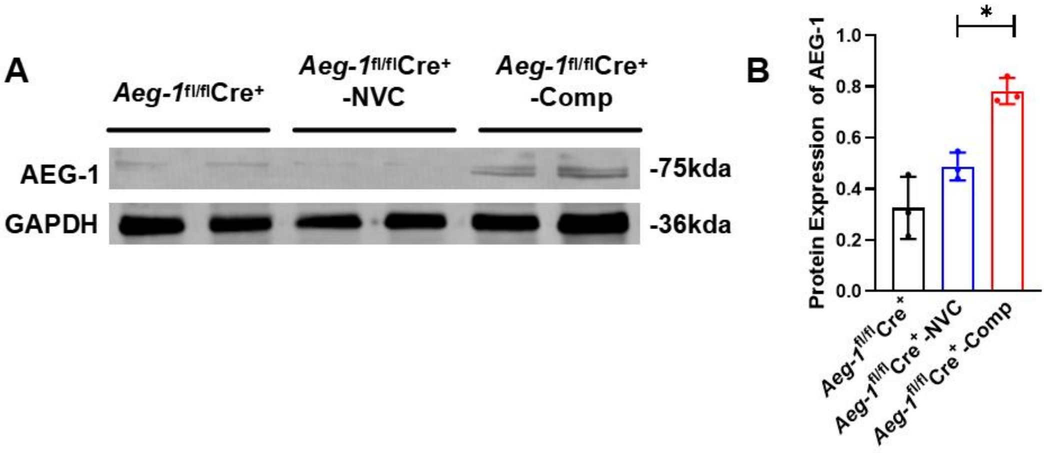

Fig. S4 Successful re-expression of *Aeg-1* in the hippocampus and neocortex of *Aeg-1*$^{\mathbf{fl/fl}}$Cre^{+} mice. (A-B) Western blotting analysis of *Aeg-1* in the hippocampus of *Aeg-1*$^{fl/fl}$Cre^{+}, *Aeg-1*$^{fl/fl}$Cre^{+}-NVC and *Aeg-1*$^{fl/fl}$Cre^{+}-Comp mice. (Results analyzed by Tukey's test. *P < 0.05).

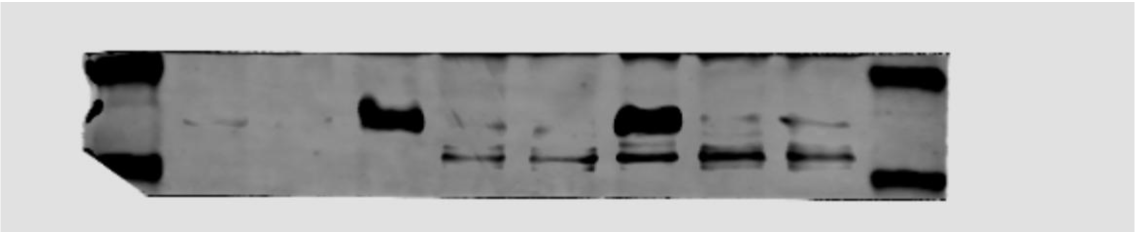

Fig. S5 (Original data) Raw images of Western blot for AEG-1 (from Fig. 3E). The lanes from left to right were marker, *Aeg-1*$^{fl/fl}$Cre^{+} hippocampus, *Aeg-1*$^{fl/fl}$Cre^{+} neocortex, *Aeg-1*$^{fl/fl}$Cre^{+} kidney, WT hippocampus, WT neocortex, WT kidney, *Aeg-1*$^{fl/wt}$Cre^{+} hippocampus, *Aeg-1*$^{fl/wt}$Cre^{+} neocortex and marker. The sizes of marker bans from top to bottom were 70 KD, 55 KD, respectively.

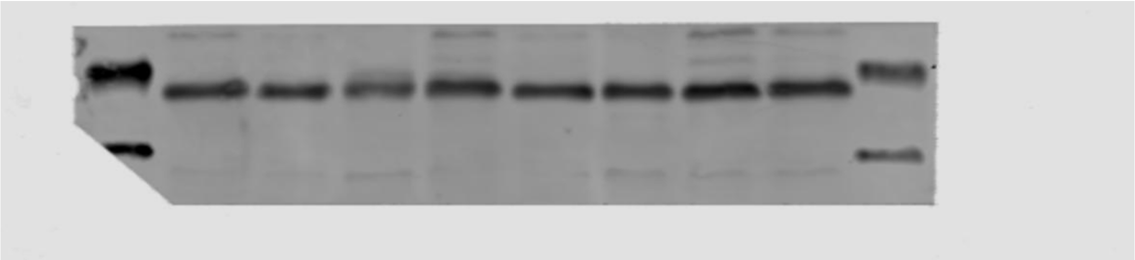

Fig. S6 (Original data) Raw images of Western blot for GAPDH (from Fig. 3E). The lanes from left to right were marker, *Aeg-1*$^{fl/fl}$Cre^{+} hippocampus, *Aeg-1*$^{fl/fl}$Cre^{+} neocortex, *Aeg-1*$^{fl/fl}$Cre^{+} kidney, WT hippocampus, WT neocortex, WT kidney, *Aeg-1*$^{fl/wt}$Cre^{+} hippocampus, *Aeg-1*$^{fl/wt}$Cre^{+}

neocortex and marker. The sizes of marker bans from top to bottom were 40 KD, 35 KD, respectively.

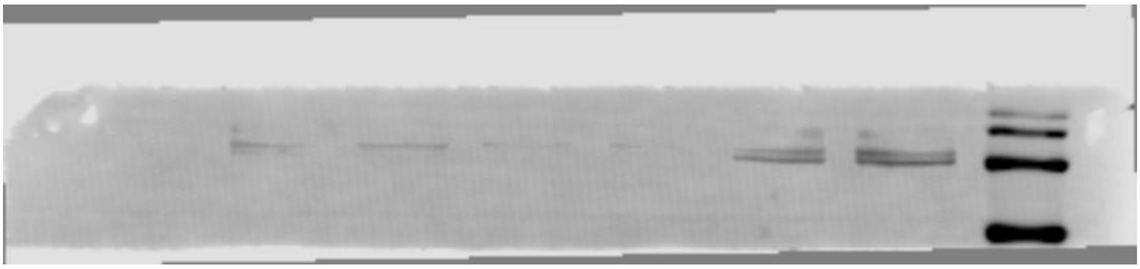

Fig. S7 (Original data) Raw images of Western blot for AEG-1 (from Fig. S4). The lanes from left to right were *Aeg-1*$^{fl/fl}$Cre^{+} hippocampus-1, *Aeg-1*$^{fl/fl}$Cre^{+} hippocampus-2, *Aeg-1*$^{fl/fl}$Cre^{+}-NVC hippocampus-1, *Aeg-1*$^{fl/fl}$Cre^{+}-NVC hippocampus-2, and *Aeg-1*$^{fl/fl}$Cre^{+}-Comp hippocampus-1, *Aeg-1*$^{fl/fl}$Cre^{+}-Comp hippocampus-2 and marker. The sizes of marker bans from top to bottom were 130 KD, 100 KD, 70 KD, 55 KD, respectively.

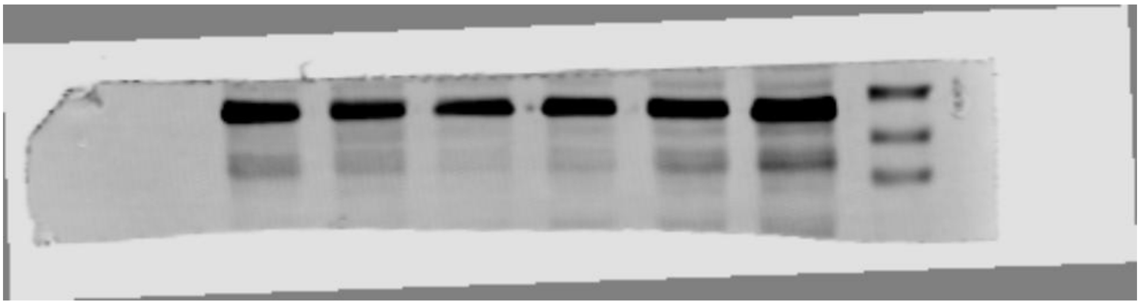

Fig. S8 (Original data) Raw images of Western blot for GAPDH (from Fig. S4). The lanes from left to right were *Aeg-1*$^{fl/fl}$Cre^{+} hippocampus-1, *Aeg-1*$^{fl/fl}$Cre^{+} hippocampus-2, *Aeg-1*$^{fl/fl}$Cre^{+}-NVC hippocampus-1, *Aeg-1*$^{fl/fl}$Cre^{+}-NVC hippocampus-2, and *Aeg-1*$^{fl/fl}$Cre^{+}-Comp hippocampus-1, *Aeg-1*$^{fl/fl}$Cre^{+}-Comp hippocampus-2 and marker. The sizes of marker bans from top to bottom were 40 KD, 35 KD, 25 KD, respectively.

Table s1 SgRNA sequence

sgRNA	sgRNA sequence（5’→3’）	PAM
5’end	CAGAGCACAAACAACAAGCT	CGG
3’end	ATGCGCATTAAAGAGTCCTT	AGG

Table s2 Primer information for genotyping *Aeg-1*$^{fl/wt}$ and *Aeg-1*$^{fl/wt}$Cre^{+} mice

NO.	Primer Name	Sequence (5'→3')	GC %	Tm (°C)	Expected Band Size	Primer Illustration
1	200244-Mtdh-ssDNA-5wt-tF1	GCAGACACTGGCTCTCAAATATATCC	46.2	56.5	fl/fl=570 bp fl/wt=570/471 bp wt/wt=471 bp	5' initial screening, detecting the wild-type across the 5' loxP sites on both ends; applicable for homozygous identification.
	200244-Mtdh-ssDNA-5wt-tR1	TCTTCATAACTGATCTGCATTTGGC	40	56.4		
2	200244-Mtdh-ssDNA-D5-5tF1	ACTAGGTTTCAGACAAGATTAGCCATG	40.7	55.5	fl=527 bp	D5-5
	common_En2-R	CCAACTGACCTTGGGCAAGAACAT	50	60.1	wt=none	
3	ZMK2F4	GCATCGCATTGTCTGAGTAGGTG	52.2	60.1	fl=534 bp	D5-3
	200244-Mtdh-ssDNA-D5-3tF1	CATGGAGTTCAGGTGCTAATACCAT	44	55.3	wt=none	
4	200244-Mtdh-ssDNA-D3-5tF1	GGTTGGAATTGACCTACAAAGTGC	45.8	55.7	fl=514 bp	D3-5
	LAR3	CACAACGGGTTCTTCTGTTAGTCC	50	55.8	wt=none	
5	Neo-3F	TCTGAGGCGGAAAGAACCAG	55	54.3	fl=499 bp	D3-3
	200244-Mtdh-ssDNA-D3-3tR1	AGTTAGCTCAACTCTGAGGCCACA	50	56.2	wt=none	
6	Cre-up	GCCTGCATTACCGGTCGATGC	50	54.6	T=481 bp	Detecting

Cre-low	CAGGGTGTTATAAGCAATCCC	53	55	wt=none	Emx1-CRE

Table s3 Probe primers of Southern blot

NO.	Primer Name	Sequence (5'→3')	GC %	Product length	Selected
Pin-1	200244-ssDNA-Mtdh-PinF1	TCTGGTTCATTGGATTCAACTATCC	36.6	462 bp	Selected
	200244-ssDNA-Mtdh-PinR1	AAAGTGCCCAACATGCAGAATGG			
Pin-2	200244-Mtdh-ssDNA-D5-5tF1	GTGGCTTGTCATTGTGTAGGCATC	36.8	456 bp	Reserved
	common_En2-R	TCTAATCCTTAGAGCAACCCCGC			

Table s4 Antibody Information Table

Antibody Name	Supplier	Catalog Number	Type	Host Species	Dilution Used	Application
Anti-AEG-1	Abcam	ab227981	Monoclonal	Rabbit	1/200	Immunofluorescence, Western Blot, Immunohistochemistry
Anti-NeuN	Abcam	ab104224	Monoclonal	Mouse	1/1000	Immunofluorescence
Anti-GFAP	Abcam	ab4674	Polyclonal	Chicken	1/600	Immunofluorescence
Anti-Iba1	Abcam	ab283319	Monoclonal	Mouse	1/100	Immunofluorescence
Goat anti rabbit IgG H&L (Alexa Fluor® 488)	Abcam	ab150077	/	Goat	1/1000	Immunofluorescence
Goat anti mouse IgG H&L (Alexa Fluor® 647)	Abcam	ab150115	/	Goat	1/1000	Immunofluorescence
Goat anti chicken IgY H&L (Alexa Fluor® 594)	Abcam	ab150172	/	Goat	1/1000	Immunofluorescence
GAPDH Monoclonal antibody	Proteintech	60004-1-Ig	Monoclonal	Mouse	1/100000	Western Blot
IRDye 800CW goat anti-rabbit IgG(H+L)	LI-COR Biosciences	926-32211	/	Goat	1/2000	Western Blot
IRDye 800CW goat anti-mouse IgG(H+L)	LI-COR Biosciences	926-32210	/	Goat	1/2000	Western Blot
